# RIP3-dependent necroptosis contributes to the pathogenesis of chronic obstructive pulmonary disease

**DOI:** 10.1172/jci.insight.144689

**Published:** 2021-06-22

**Authors:** Dongshi Chen, Alyssa D. Gregory, Xiaoyun Li, Jianxin Wei, Christine L. Burton, Gregory Gibson, Stephen J. Scott, Claudette M. St. Croix, Yingze Zhang, Steven D. Shapiro

**Affiliations:** 1 Division of Pulmonary, Allergy and Critical Care Medicine, Department of Medicine, and; 2 Department of Cell Biology, School of Medicine, University of Pittsburgh, Pittsburgh, Pennsylvania, USA.

**Keywords:** Cell Biology, Pulmonology, Apoptosis, COPD, Macrophages

## Abstract

Necroptosis has emerged as a potential mechanism in the pathogenesis of chronic obstructive pulmonary disease (COPD). Here, we found that markers of necroptosis, including high mobility group box 1 release and phosphorylation of mixed lineage kinase domain-like protein (p-MLKL), were markedly induced in the late stage of cigarette smoking–induced (CS-induced) emphysema in mouse lung tissue as well as in lung epithelial cells and organoids with higher dosage of or more prolonged exposure to cigarette smoking extract (CSE). Apoptotic signals were also detected and maximally induced in the early stage of CS-exposed mice and CSE-treated epithelial cells. Inhibition of apoptosis by Z-VAD, a pan-caspase inhibitor, switched the cellular stress to enhanced necroptosis in lung epithelial cells and organoids treated with CSE. Depletion or inhibition of receptor-interacting protein kinase 3 (RIP3) or MLKL attenuated the CSE-induced cell death, suggesting that necroptosis contributes to CSE-induced cell death. Silencing or inhibition of RIP1 had no protective effect, indicating a RIP1-independent RIP3 activation pathway. CSE-induced necroptosis released more damage-associated molecular patterns and evoked greater engulfment but slower clearance by bone marrow–derived macrophages, leading to enhanced expression of proinflammatory cytokines *Tnf****α* and *Il6*. Finally, our in vivo data verified that inhibition of necroptosis by RIP3 inhibitor GSK’872 protected mice from CS-induced emphysema and suppressed the lung inflammation. In conclusion, we provide evidence that necroptosis contributes to the pathogenesis of COPD. Targeting RIP3 and its downstream pathway may be an effective therapy for COPD.

## Graphical abstract

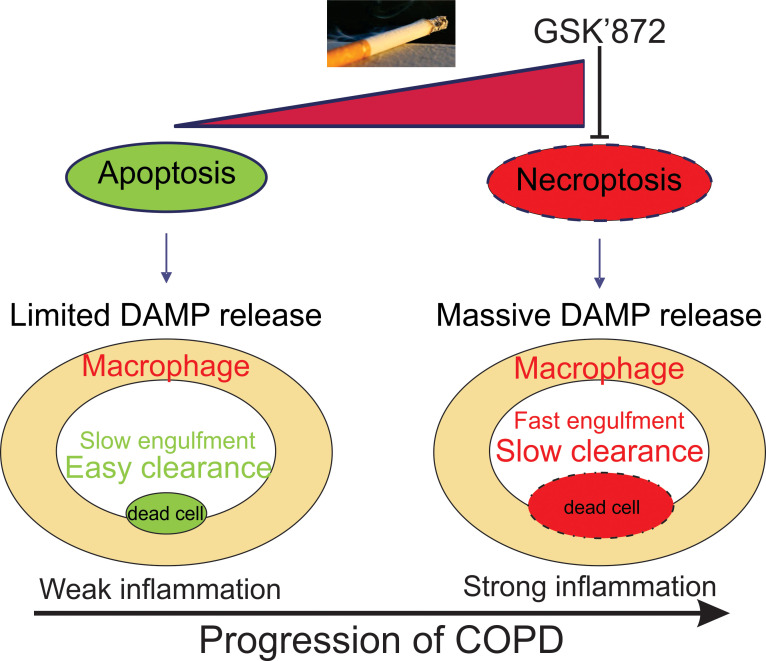

## Introduction

Chronic obstructive pulmonary disease (COPD) affects more than 10% of the world population over the age of 40 years and ranks as the fourth leading cause of death worldwide (1, 2). The clinical phenotype of COPD includes emphysema and chronic bronchitis (3). Emphysema is defined as enlarged airspaces as a result of inflammation, cell death, and elastic fiber destruction, with inadequate repair (4). The identification of molecular mediators that trigger lung responses involving inflammation and alveolar cell death will help develop novel therapies for COPD. Cigarette smoking (CS) is the most significant risk factor for COPD (5). The airway epithelial layer forms the first physical barrier to inhaled harmful substances, and epithelial cell death plays an essential regulatory role in the subsequent proinflammatory response (6). Exposure to CS has been reported to induce epithelial cell death by apoptosis and necroptosis, which also occur in patients with COPD (7, 8). Compared with apoptosis, necroptosis is considered a type of programmed cell death involving greater damage-associated molecular pattern (DAMP) release and inflammatory response (9, 10). Necroptosis is a newer concept of programmed cell death, which is driven by receptor-interacting protein kinase 1/3 (RIP1/3) and mixed lineage kinase domain-like protein (MLKL) molecular pathway (11, 12). The release of inflammatory substances in necroptosis contributes to persistent lung inflammation, which cannot be explained by apoptosis-derived cell death. There is accumulating evidence that necroptosis occurs in patients with COPD (8, 13), but its role in inflammation and disease pathogenesis is unknown.

In this study, we show that the necroptosis markers phosphorylated MLKL (p-MLKL) and high mobility group box 1 (HMGB1) release are markedly elevated in patients with COPD, smoking-exposed mice, and cigarette smoking extract–treated (CSE-treated) lung epithelial cells. Although apoptosis was also observed, CSE-induced necroptosis triggered enhanced phagocytosis and production of inflammatory mediators by macrophages, which is a major source of lung inflammation in COPD (6, 14). Inhibition or depletion of RIP3 suppressed the CSE-induced necroptosis in vitro, and inhibition of RIP3 attenuated the emphysema caused by CS in vivo. Therefore, amelioration of RIP3 activation in CS-induced necroptosis may limit progression of COPD.

## Results

### Both apoptosis and necroptosis are induced in human COPD and mice with smoke exposure.

**Figure 1:**
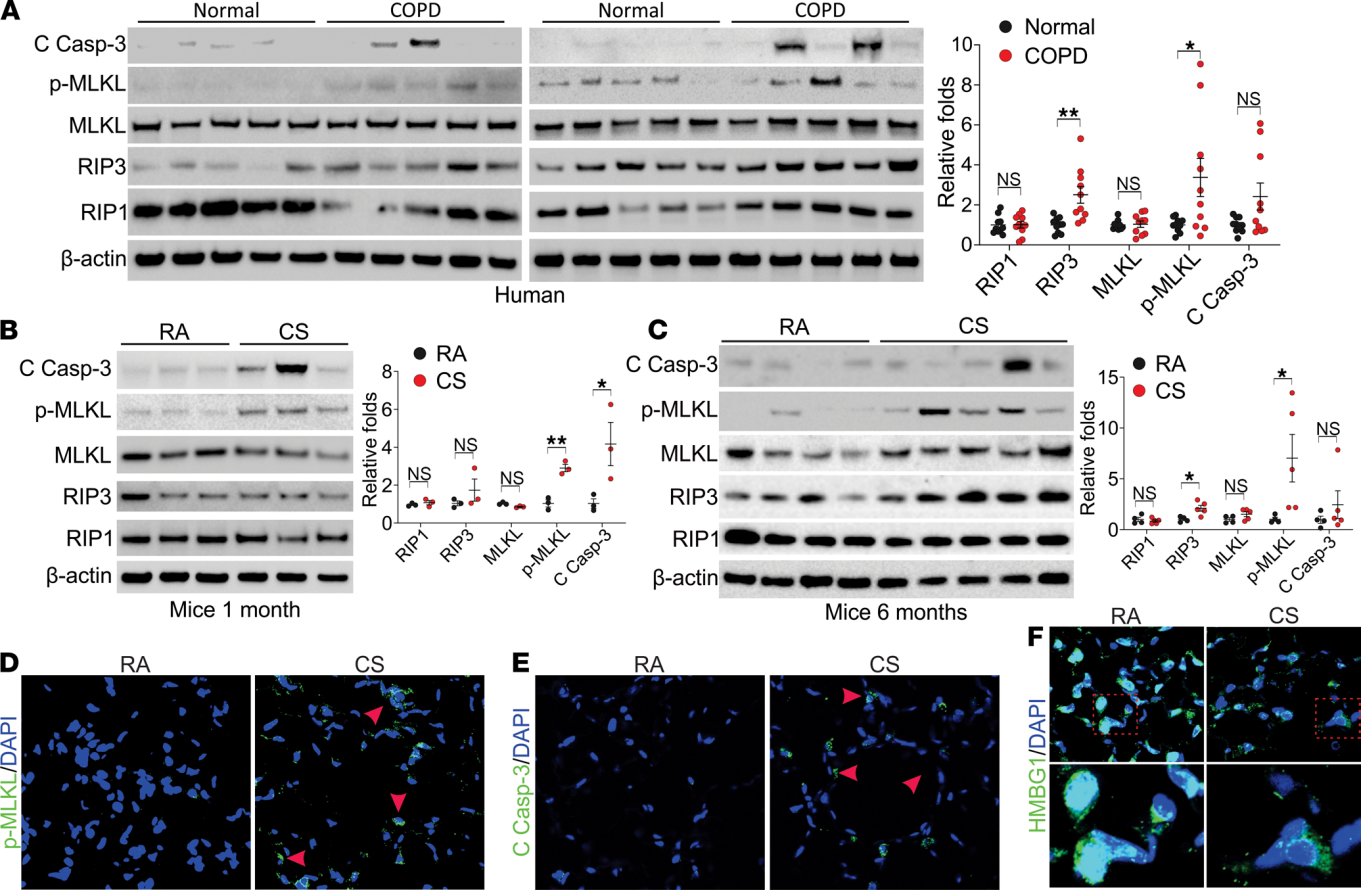
Necroptosis and apoptosis were detected in the lung tissue from COPD patients and CS treated mice. (**A**) Western blot analysis of cleaved caspase-3 (C Casp-3), p-MLKL (S358, p-MLKL), MLKL, RIP3, and RIP1 in the lung tissue from donors and patients with COPD. *Left*, the representative pictures; *right*, the quantification of each protein’s expression. (**B** and **C**) Western blot analysis of C Casp-3, p-MLKL (S345, p-MLKL), MLKL, RIP3, and RIP1 in the lung tissue from C57BL/6J mice exposed to room air (RA) or CS for 1 month (**B**) and 6 months (**C**). *Left*, the representative pictures; *right*, the quantification of each protein’s expression. (**D**–**F**) Immunofluorescence staining of p-MLKL (**D**), C Casp-3 (**E**), and HMGB1 (**F**) in lung tissue from mice exposed to RA or CS for 6 months. Each experiment was repeated 3 times. Original magnification, ×400 (**D** and **E**), ×600 (**F**). Red arrowheads indicate the immunofluorescence-positive cells. Data represent the means ± SEM. *, *P* < 0.05; **, *P* < 0.01. Student’s *t* test was conducted for each 2-group comparison.

To study cell death signals in COPD, we examined the expression of RIP1, RIP3, MLKL p-MLKL, and cleaved caspase-3 in the lungs of donors and COPD patients (*n* = 10 for each group). We found significant upregulation of RIP3 and p-MLKL and occasional induction of cleaved caspase-3 in patients with COPD (Figure 1A), indicating that strong necroptosis and some apoptosis coexisted in patients with COPD. To confirm the apoptosis and necroptosis observed in COPD lungs, we analyzed the expression of related proteins in the lung tissues from C57BL/6J mice with 1 month and 6 months of cigarette smoke exposure and nonsmoking controls. Both p-MLKL and cleaved caspase-3 were significantly induced upon 1 month of smoking (Figure 1B). Continued upregulation of RIP3 and p-MLKL and blunted induction of cleaved caspase-3 was found in mice with 6 months’ smoking where emphysema was observed (15) (Figure 1C). Similarly, mild induction of microtubule-associated protein 1A/1B-light chain 3 (LC3), the autophagy marker, was found in the early stage of CS exposure but absent in the mice with 6 months of smoking (Supplemental Figure 1, A and B; supplemental material available online with this article; https://doi.org/10.1172/jci.insight.144689DS1). Consistent with immunoblot analysis, increased p-MLKL and cleaved caspase-3 immunostaining was observed in epithelial cells after 6 months of CS exposure (Figure 1, D and E). The release of HMGB1 into the cytosol, one of the significant necroptosis markers (16), was also detected in the lung tissues from mice with 6 months of smoke exposure (Figure 1F). These results suggest that the continued induction of necroptosis over time might contribute to the pathogenesis of COPD.

### Cigarette smoke extract induces both apoptosis and necroptosis in vitro.

**Figure 2:**
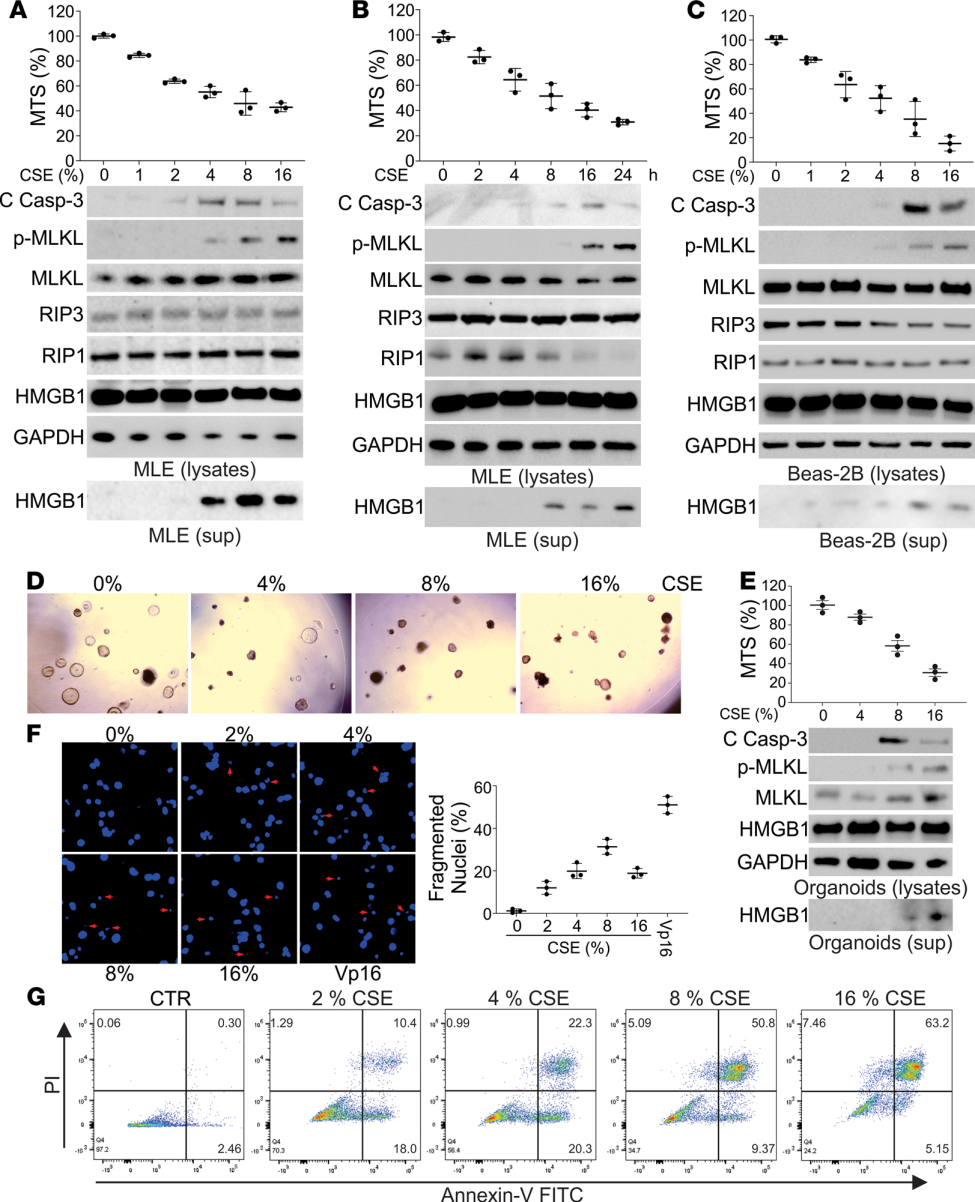
Necroptosis and apoptosis were detected in the CSE-treated lung epithelial cells and organoids. (**A**) MLE-12 cells were treated with the indicated concentration of CSE for 16 hours. *Upper*, the cell viability was analyzed by MTS assay; *lower*, the expression of indicated proteins in cell lysates and HMGB1 in cell supernatant was analyzed by Western blot. (**B**) MLE-12 cells were treated with 8% CSE for the indicated time. *Upper*, the cell viability was analyzed by MTS assay; *lower*, the expression of indicated proteins in cell lysates and HMGB1 in cell supernatant was analyzed by Western blot. (**C**) Beas-2B cells were treated with the indicated concentration of CSE for 16 hours. *Upper*, the cell viability was analyzed by MTS assay; *lower*, the expression of indicated proteins in cell lysates and HMGB1 in cell supernatant was analyzed by Western blot. (**D**) The organoids derived from mouse lungs were treated with indicated concentrations of CSE for 16 hours. The representative pictures of organoids were shown. (**E**) The organoids were treated as in **D**. *Upper*, the cell viability was analyzed by MTS assay; *lower*, the expression of indicated proteins in cell lysates and HMGB1 in cell supernatant was analyzed by Western blot. (**F**) The nuclei fragmentation of MLE-12 cells treated with the indicated concentration of CSE for 16 hours was analyzed using Hoechst 33258 staining. Etoposide (Vp-16) (25 μM) was used as a positive control. *Left*, the representative pictures; *right*, the percentage of fragmented nuclei was counted. Original magnification, ×600. Arrow indicates the fragmented nuclei. (**G**) The apoptosis and necroptosis of MLE-12 cells treated with indicated concentrations of CSE were analyzed by annexin V/PI staining followed by flow cytometry analysis. The representative data were shown. Data represent the means ± SEM. Each experiment was repeated 3 times. PI, propidium iodide.

To further delineate necroptosis and apoptosis with smoking, we next investigated cells exposed to CSE in vitro. We observed that MLE-12 cells treated with CSE had decreased cell viability in both a dose- and time-dependent manner (Figure 2, A and B). Phosphorylated-MLKL in the cells and release of HMGB1 in cell culture medium, but not HMGB1 in the cells, were also induced by CSE, increasing with both dose and time of exposure (Figure 2, A and B). In contrast, the expression of cleaved caspase-3 peaked at 8% CSE treatment for 16 hours but dropped at the higher dosage or more extended treatment time point (Figure 2, A and B). The induction of LC3 happened in much lower dosages (1%~4%) of CSE treatment in MLE-12 cells (Supplemental Figure 1C), indicating CSE induced autophagy at the very beginning. Inhibition of autophagy by 3-methyladenine (3 MA) did not change the higher dosages (4%~8%)of CSE-induced apoptosis or necroptosis (Supplemental Figure 1D), indicating that early induction of autophagy did not involve cell death at the later stage of CSE exposure. CSE treatment also led to the loss of cell viability and escalated expression of p-MLKL and HMGB1 release in Beas-2B cells, a human lung epithelial cell line (Figure 2C), and organoids derived from mouse lung tissues (Figure 2, D and E). Consistent with the upregulation of caspase-3 cleavage, nuclear fragmentation, a morphological hallmark of apoptosis, was maximally induced by 8% CSE in MLE-12 cells (Figure 2F). Flow cytometry analysis revealed that CSE treatment induced early apoptosis of MLE-12 cells (annexin V^+^PI^–^ cells) that was decreased with greater than 4% CSE (Figure 2G). CSE treatment continuously increased MLE-12 PI^+^ cells, which included necroptotic and late apoptotic cells (Figure 2G). To further confirm our data, the human bronchial epithelial cells (HBEs) from normal lung and the lungs of COPD patients were used. Our results showed that HBEs from COPD patients were more susceptible to CSE-induced cell loss, with higher induction of p-MLKL but less cleavage of caspase-3 (Supplemental Figure 2A). Collectively, our results indicate that both apoptosis and necroptosis were triggered by CSE treatment in vitro, and increased cellular stress by CSE exposure shifted the cell death from early apoptosis to late apoptosis and eventually to necroptosis.

### CSE-induced apoptosis inhibits necroptosis.

**Figure 3:**
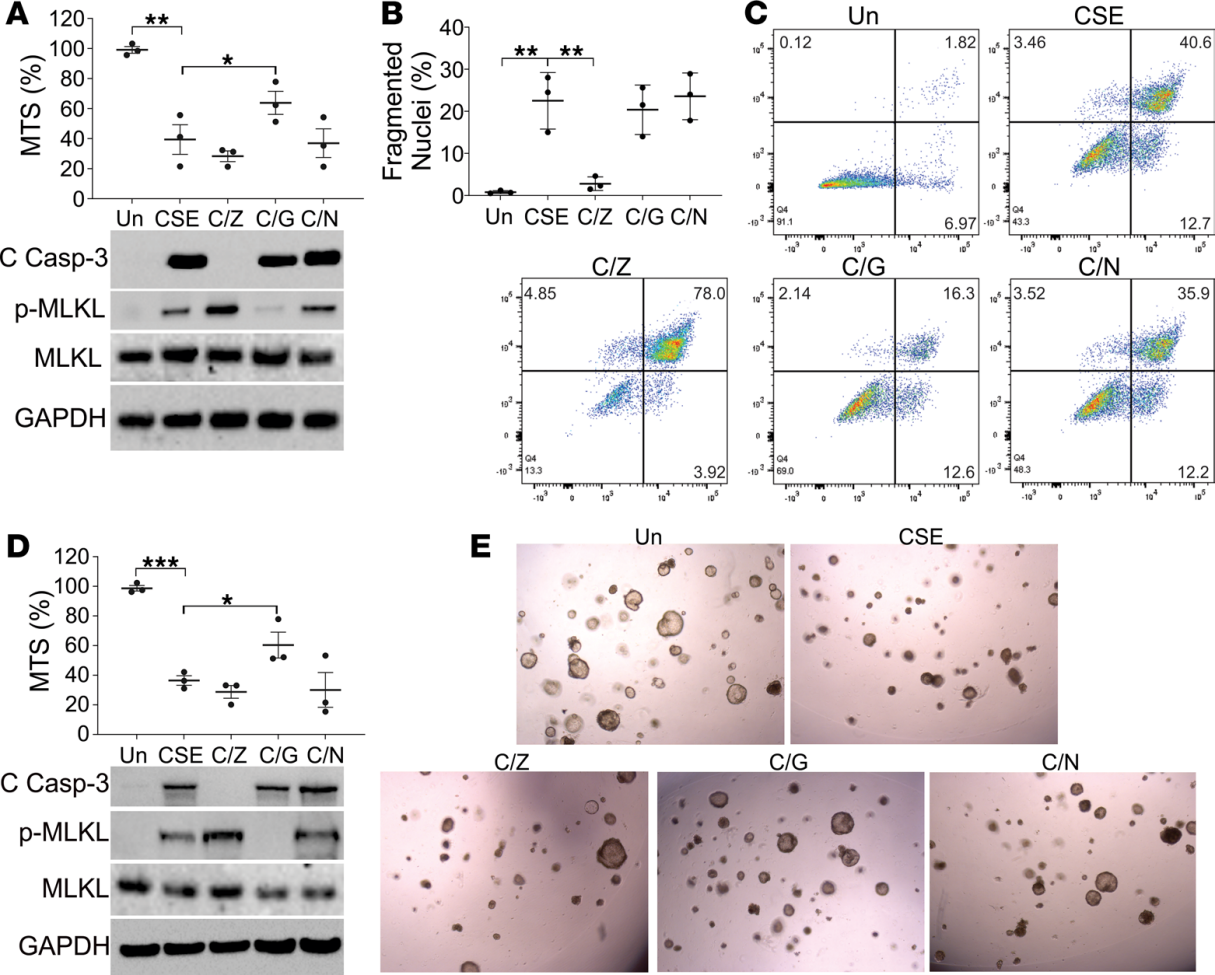
Inhibition of CSE-induced apoptosis enhanced necroptotic stress. (**A**) MLE-12 cells pretreated with 10 μM Z-VAD (Z), 5 μM GSK’872 (G), or 10 μM necrostatin-1 (N) were treated with 8% CSE for 16 hours. *Upper*, the cell viability was analyzed by MTS assay; *lower*, the expression of indicated proteins in cell lysates was analyzed by Western blot. (**B**) The nuclei fragmentation of MLE-12 cells treated as in **A** was analyzed using Hoechst 33258 staining. (**C**) The apoptosis and necroptosis of MLE-12 cells treated as in **A** were analyzed by annexin V/PI staining followed by flow cytometry analysis. The representative data were shown. (**D**) The organoids derived from mouse lung were treated with as in **A**. *Upper*, the cell viability was analyzed by MTS assay; *lower*, the expression of indicated proteins in cell lysates was analyzed by Western blot. (**E**) The representative pictures of organoids treated as in **A**. Each experiment was repeated 3 times. Data represent the means ± SEM. *, *P* < 0.05; **, *P* < 0.01; ***, *P* < 0.001. One-way ANOVA with Tukey’s multiple-comparison test was conducted.

The maximal induction of apoptosis and necroptosis in different stages of CSE treatment suggested the switch of apoptosis and necroptosis. Pretreating MLE-12 cells with Z-Val-Ala-DL-Asp (OMe)-fluoromethylketone (Z-VAD; Z) did not attenuate CSE-induced cell death (Figure 3A), even though it had its expected effect of inhibiting apoptosis with suppression of cleaved caspase-3 (Figure 3A), fewer fragmented nuclei (Figure 3B), and reduction of early (annexin V^+^PI^–^) apoptotic cells (Figure 3C). In the absence of apoptosis, CSE-induced necroptosis was enhanced, as indicated by p-MLKL expression (Figure 3A) and PI^+^ cells analyzed by flow cytometry (Figure 3C). Inhibition of RIP1 with necrostatin (Nec-1, N) did not affect the total cell death, apoptosis, or necroptosis induced by CSE (Figure 3, A–C). In contrast, pretreatment with GSK’872, a RIP3 inhibitor, partially protected the MLE-12 cells from CSE-induced cell loss (Figure 3A), and suppressed p-MLKL (Figure 3A) and PI^+^ cells (Figure 3C), with no effect on markers of apoptosis (Figure 3, A and B). Pretreatment with Z-VAD, GSK’872, and Nec-1 had similar effects in organoids exposed to CSE as in MLE-12 cells (Figure 3, D and E). GSK’872, rather than Z-VAD, also attenuated CSE-induced cell loss and p-MLKL signal in HBEs (COPD) (Supplemental Figure 2B). Therefore, our data suggest that inhibition of apoptosis enhanced CSE-induced necroptosis, which was RIP3, but not RIP1, dependent.

### RIP3/MLKL is essential for necroptosis induced by CSE.

**Figure 4:**
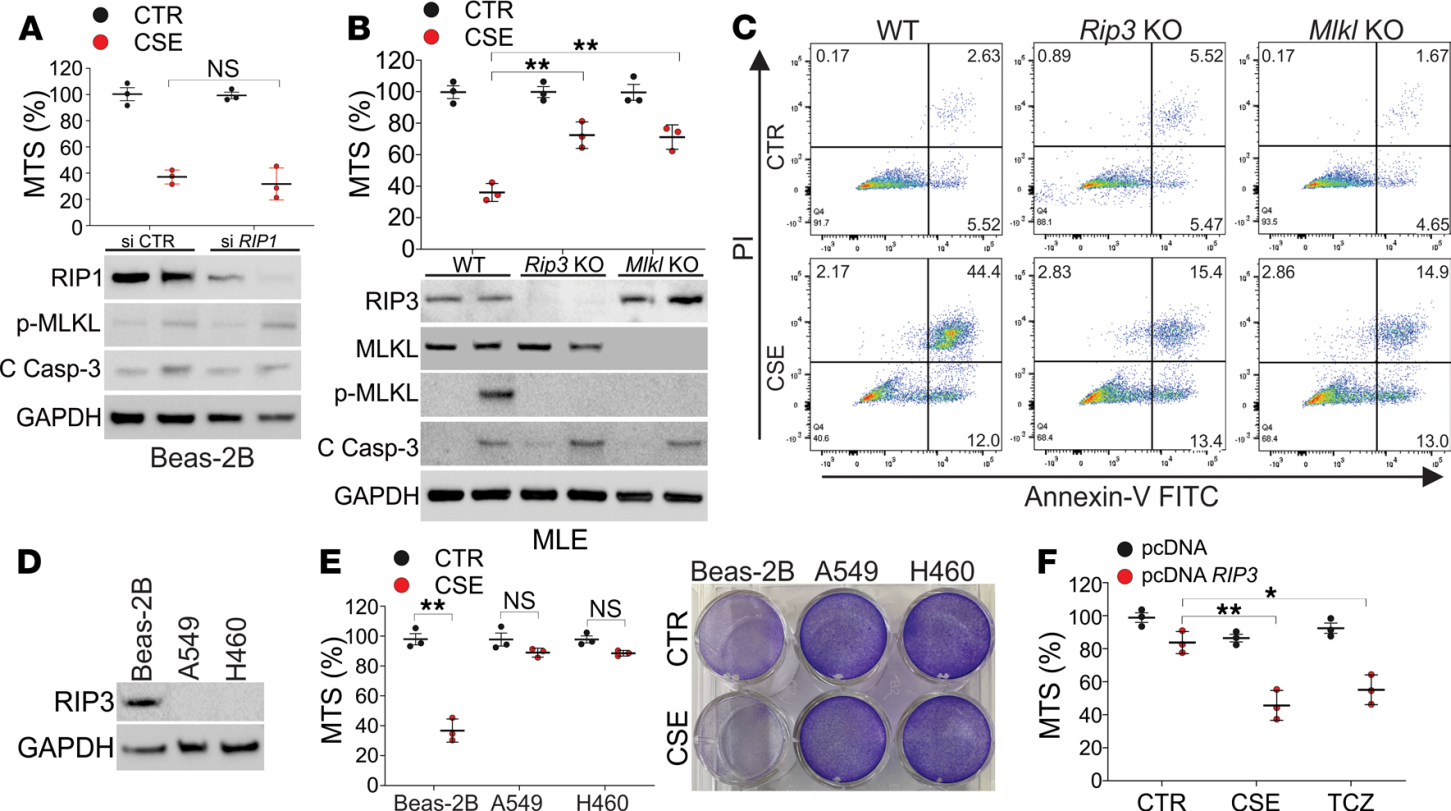
CSE-induced necroptosis is mediated by RIP3/MLKL. (**A**) Beas-2B cells transfected with control or RIP1 siRNA were treated with 8% CSE for 16 hours. *Upper*, the cell viability was analyzed by MTS assay; *lower*, the expression of indicated proteins in cell lysates was analyzed by Western blot. (**B**) WT, *RIP3*-KO, and *MLKL*-KO CRISPR/Cas9-deleted MLE-12 cells were treated with 8% CSE for 16 hours. *Upper*, the cell viability was analyzed by MTS assay; *lower*, the expression of indicated proteins in cell lysates was analyzed by Western blot. (**C**) The apoptosis and necroptosis of WT, *RIP3*-KO, and *MLKL*-KO MLE-12 cells treated as in **B** were analyzed by annexin V/PI staining followed by flow cytometry analysis. The representative data were shown. (**D**) The expression of RIP3 in Beas-2B, A549, and H460 cells. (**E**) Beas-2B, A549, and H460 cells were treated with 8% CSE for 16 hours. The cell viability was analyzed by MTS assay (*left*) and crystal violet staining (*right*). (**F**) A549 cells transfected with control (pcDNA3.1) or RIP3 (pcDNA3.1 V5-*RIP3*) plasmids were treated with 8% CSE or 20 ng/mL TNF-α+1 μg/mL cycloheximide+10 μM Z-VAD (TCZ) for 16 hours. The cell viability was analyzed by MTS assay. Each experiment was repeated 3 times. Data represent the means ± SEM. NS, *P* > 0.05; *, *P* < 0.05; **, *P* < 0.01. Two-way ANOVA with Tukey’s multiple-comparison test was conducted (**A**, **B**, and **F**). Student’s *t* test was conducted for each 2-group comparison (**E**).

To further verify the importance of RIP3 but not RIP1 in CSE-induced necroptosis, we first knocked down RIP1 in Beas-2B cells by siRNA and found that the absence of RIP1 did not affect CSE-induced cell death, activation of MLKL, or cleavage of caspase-3 (Figure 4A). To confirm the role of RIP3 in CSE-induced necroptosis in lung cells, we generated *Rip3*-KO MLE-12 cells by CRISPR/Cas9. Depletion of *Rip3* suppressed necroptosis induced by CSE in MLE-12 cells but did not affect apoptosis (Figure 4, B and C). Since MLKL is the downstream effector of RIP3 and the final executor of necroptosis (12), we further investigated the role of MLKL in CSE-induced necroptosis. The depletion of *Mlkl* by CRISPR/Cas9 also protected MLE-12 cells from CSE-induced necroptosis but not apoptosis (Figure 4, B and C). RIP3 is not significantly expressed in many cell lines, especially in cancer cells (17). We analyzed A549 and H460 cells and found that they both had relatively lower expression of RIP3 (Figure 4D) and did not have any response to CSE-induced cell death (Figure 4E). Enhanced expression of RIP3 in A549 cells by transfection of a *RIP3* expression vector sensitized them to CSE-induced cell death, as did a traditional necroptosis inducer, a mixture of TNF-α, cycloheximide, and Z-VAD (Figure 4F). Collectively, our results suggest that CSE-induced necroptosis in lung epithelial cells is mediated by RIP3/MLKL, rather than RIP1.

### CSE-induced necroptosis promotes inflammation.

**Figure 5:**
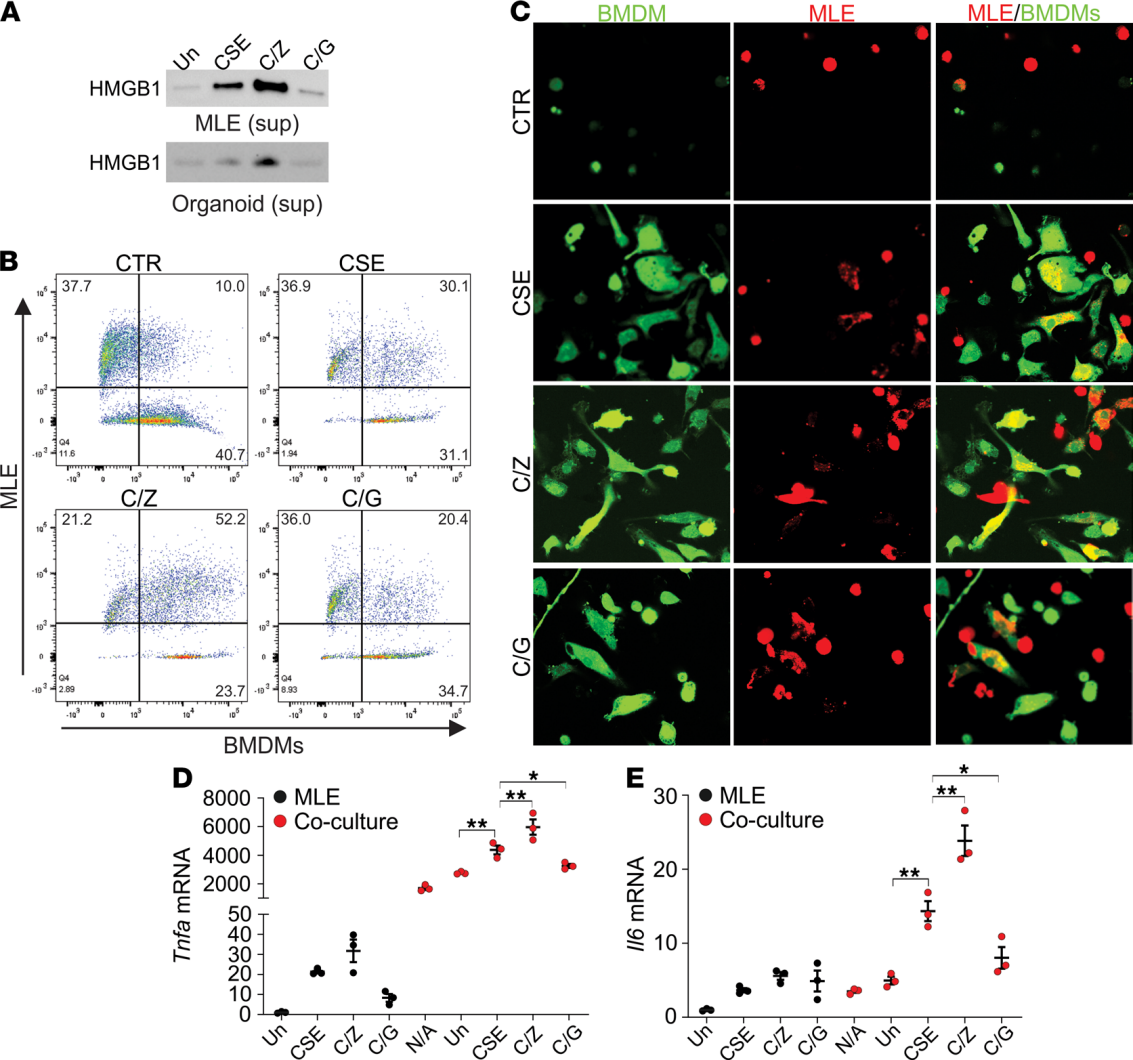
CSE-induced necroptosis is the major source of inflammation. (**A**) The HMGB1 release in the supernatant medium in MLE-12 (*upper*) and organoids (*lower*) treated with 8% CSE in combination with 10 μM Z-VAD (Z) or 5 μM GSK’872 (G) for 16 hours. (**B** and **C**) MLE-12 cells were treated as in **A** and stained with CellTrace Far Red (red), then cocultured with BMDMs stained with CellTrace CFSE (green) for 8 hours. BMDM phagocytosis was analyzed by flow cytometry (**B**) and confocal microscopy (**C**). Representative phagocytosis was shown. Original magnification, ×1000. (**D** and **E**) MLE-12 cells treated as in **B** were cocultured with BMDMs. The mRNA level of TNF-α (**D**) and IL-6 (**E**) in MLE-12 cells or MLE-12 and BMDM coculture system was analyzed. Each experiment was repeated 3 times. Data represent the means ± SEM. *, *P* < 0.05; **, *P* < 0.01. One-way ANOVA with Tukey’s multiple-comparison test was conducted. N/A, untreated BMDMs.

COPD is characterized by chronic inflammation in the lung that results in progressive and irreversible airflow obstruction (14, 18). Apoptosis is thought to be antiinflammatory, or at least not promote inflammation, while necroptosis leads to robust inflammation (19). The release of HMGB1, S100A4, and ATP, the critical DAMP markers, in CSE-treated MLE-12 cells or organoids was enhanced by Z-VAD but suppressed by GSK’872 pretreatment (Figure 5A and Supplemental Figure 3, A and B), indicating higher DAMP release by CSE induced necroptosis. When CSE-treated MLE-12 cells were cocultured with bone marrow–derived macrophages (BMDMs), we observed marked increases in the engulfment of dying MLE-12 cells by BMDMs (Figure 5, B and C, and Supplemental Figure 3C). Phagocytosis of CSE-treated MLE-12 cells by BMDMs was compromised by pretreatment with GSK’872 but enhanced by pretreatment with Z-VAD (Figure 5, B and C, and Supplemental Figure 3C). A similar scenario was observed in the MLE-12 and alveolar macrophage (AM) coculture system (Supplemental Figure 3D). However, the live video of the phagocytosis showed that BMDMs more efficiently digested dead MLE-12 cells treated with CSE in combination with GSK’872, as compared with those cells treated with CSE in combination with Z-VAD (Supplemental Videos 1 and 2). These data indicated that CSE-induced necroptotic cells were more difficult to clear (i.e., degrade) by BMDMs, even though necroptosis triggered more engulfment by BMDMs. The mRNA expression of proinflammatory cytokines *Tnfα* and *Il6* significantly increased in BMDMs cocultured with CSE-treated MLE-12 cells in comparison with BMDMs cocultured with nontreated MLE-12 cells or alone (Figure 5, D and E). The increased expression of *Tnfα* and *Il6* in BMDMs cocultured with CSE-treated MLE-12 cells was enhanced by Z-VAD but suppressed by GSK’872 pretreatment (Figure 5, D and E). Together, these in vitro results indicated that CSE-induced necroptosis is more proinflammatory and likely contributes to lung damage in COPD.

### Inhibition of necroptosis by RIP3 inhibitor suppresses the CS-induced COPD in mice exposed to long-term cigarette smoke.

**Figure 6:**
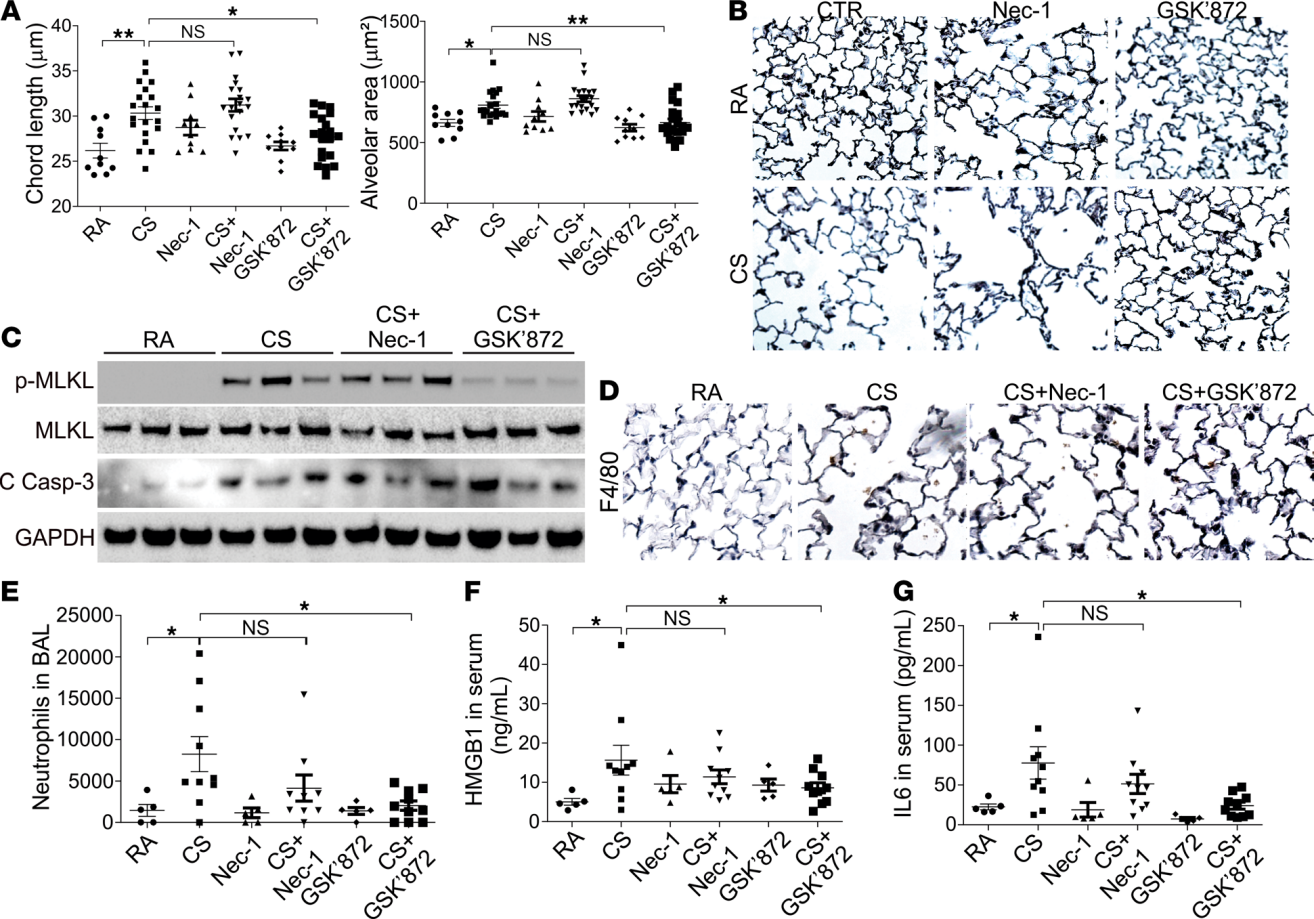
RIP3-dependent necroptosis contributes to airspace enlargement during CS exposure in vivo. C57BL/6J mice were exposed to RA (*n* = 5 for each group) or CS (*n* = 10 for each group) for 2 months and injected with control vehicle (4% DMSO), Nec-1 (5 mg/kg), or GSK’872 (0.75 mg/kg) 1 hour before CS exposure for an additional 4 months. The experiment was repeated twice. (**A**) Chord lengths (*left*) and alveolar area (*right*) of mouse lungs in indicated groups were calculated. (**B**) Representative pictures of lung. Original magnification, ×200. (**C**) The expression of indicated proteins in the lungs from different groups of mice. (**D**) The representative immunochemistry staining of F4/80 in the lungs from different groups of mice. Original magnification, ×400. (**E**) The neutrophil number in the BAL from different groups of mice. (**F** and **G**) The HMGB1 (**F**) and IL-6 (**G**) level in the serum of mice from different groups. Data represent the means ± SEM. *, *P* < 0.05; **, *P* < 0.01. One-way ANOVA with Tukey’s multiple-comparison test was conducted.

Because the CSE exposure system does not contain any nonaqueous constituents and cannot mimic the in vivo conditions for the gas-to-liquid compound exchange, we further validated the role of necroptosis in emphysema by exposing the mice to CS for 6 months. WT C57BL/6J mice exposed to CS had significant airspace enlargement in the lung when compared with RA-treated controls (Figure 6, A and B). After 2 months of CS exposure, GSK’872 was administered for an additional 4 months, and this suppressed airspace enlargement in the CS group with no effect on lung morphology in the RA group (Figure 6, A and B). In contrast, injection of Nec-1 with the same treatment schedule did not protect the mice from airspace enlargement by CS but mildly increased the airspace in both CS and RA treatment groups (Figure 6, A and B). The induction of p-MLKL in the CS group was reduced by GSK’872 injection but not affected by Nec-1 injection (Figure 6C). Regarding apoptosis, neither GSK’872 nor Nec-1 had any effects on the cleaved caspase-3 induction by CS (Figure 6C). We next investigated the impact of inhibiting necroptosis by GSK’872 on the lung inflammation induced by CS. Macrophage accumulation by F4/80 staining in the lungs of mice exposed with 6 months’ CS was reduced by GSK’872 pretreatment but not Nec-1 (Figure 6D). Administration of GSK’872 also attenuated CS-induced accumulation of neutrophils in both bronchoalveolar lavage (BAL) and lung tissues (Figure 6E and Supplemental Figure 4, A and B), as well as CD8^+^ T cells in the lung tissues (Supplemental Figure 4, A and C). CS exposure also enhanced the secretion of HMGB1 and IL-6 in the serum of treated mice, which was suppressed by GSK’872 pretreatment (Figure 6, F and G). Collectively, our results suggested that RIP3-mediated necroptosis contributes to lung inflammation and airspace enlargement in COPD.

## Discussion

**Figure 7:**
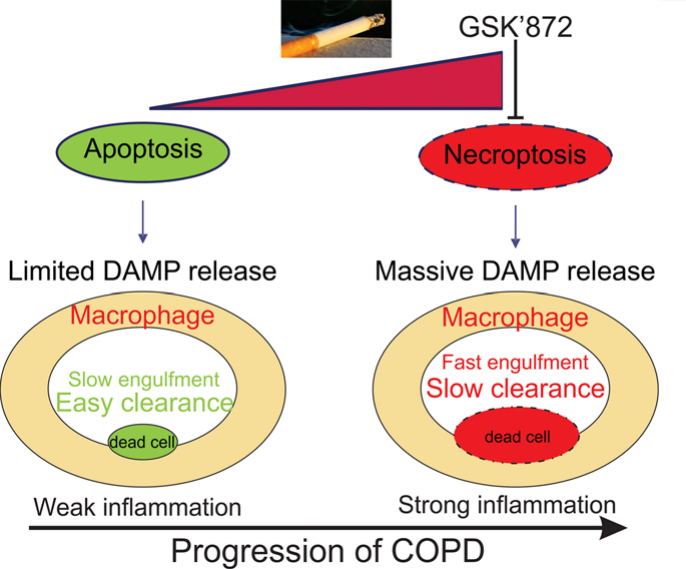
A proposed mechanism of cigarette smoke–induced necroptosis contributing to lung inflammation and COPD progression.

There is an appreciation for the role of cell death in COPD (8, 13), but it remains unclear how different forms of programmed cell death contribute alone and in combination to COPD. Our current study confirms that both apoptosis and necroptosis can be detected in the lungs of human COPD patients, in CS-treated mice, and in lung epithelial cells exposed to CSE. Importantly, necroptosis appears to play a central role in COPD pathogenesis. Using CSE-treated lung epithelial cells or organoids, we found that the CSE-induced necroptosis depended on RIP3/MLKL, not RIP1. Moreover, inhibition of RIP3 by GSK’872 attenuated inflammation and subsequent emphysema in mice exposed to 6 months’ smoking, indicating that targeting the necroptosis signaling pathway might be a potential therapeutic strategy for patients with COPD (Figure 7).

Apoptosis and necroptosis are 2 primary forms of regulated cell death, which commonly coexist (20, 21). Here, we show that CS-induced epithelial cell death switched from apoptosis to necroptosis with increasing exposure time and dosage both in vivo and in vitro, indicating different functions of apoptosis and necroptosis in different stages of COPD progression. Although autophagy was also found in the very early stage of CS exposure, it did not contribute to apoptosis and necroptosis in the later stages (Supplemental Figure 1). Epithelial cell apoptosis is a hallmark feature and one of the initial steps in the process of COPD. Induction of apoptosis can lead to airspace enlargement in several models of emphysema, some of which could be prevented by caspase inhibitors (22–25). However, there has been no evidence that inhibition of apoptosis can limit emphysema in models induced by the etiologic agent, cigarette smoke. Previously, it has been shown that apoptosis-induced airspace enlargement represents temporary acute lung injury with both alveolar collapse with tethering and enlargement of adjacent airspaces but not irreversible emphysema (18).

The current study further revealed that apoptotic signals in mouse lungs and lung epithelial cells and organoids were induced at a lower dosage and earlier time of CS treatment (Figure 1B and Figure 2), indicating the apoptosis might happen in the early stage of COPD. However, with the increase of CSE dosage or exposure time in vitro, or CS exposure time in vivo, the cellular stress switched cell death from apoptosis into necroptosis (Figure 1C and Figure 2). Moreover, the apoptosis inhibitor, Z-VAD, did not suppress CSE-induced cell death but enhanced the necroptosis in our in vitro experimental conditions. In contrast, inhibition or depletion of the necroptosis modulator, RIP3 or MLKL, suppressed CSE-induced cell death, indicating that CSE-induced cell death is partially attributable to necroptosis. Inhibition of RIP3 2 months after CS exposure slowed down emphysema development in mice, further suggesting that necroptosis contributes to the later stage of COPD that is associated with lung destruction. Hence, both apoptosis and necroptosis are both observed in COPD and perhaps intertwined in the final pathway of lung destruction with airspace enlargement.

The mode of programmed cell death may also affect efferocytosis, the ability of professional phagocytes to remove dead cells as well as subsequent pro- or antiinflammatory effects by the phagocyte. The efficiency of macrophages to phagocytize necroptotic versus apoptotic cells remains controversial (26, 27), but it is generally believed that apoptotic cells are more efficiently removed and do not provoke inflammation, whereas necroptotic cells are less efficiently cleared and stimulate inflammation (9, 19, 28, 29) with greater secretion of proinflammatory cytokines IL-6 and TNF-α (27). Here we show that inhibition of CSE-induced apoptosis by Z-VAD enhanced the uptake of dead cells but had lower efficiency of clearance (degradation) by macrophages. Inhibition of CSE-induced apoptosis by Z-VAD also enhanced the expression of proinflammatory cytokines (TNF-α and IL-6) by macrophages, which was suppressed by inhibition of necroptosis. Necroptosis also results in a massive release of the DAMPs, including S100 proteins, ATP, and HMGB1 (30). Elevated HMGB1 expression is inversely correlated to forced expiratory volume in COPD patients (31) and sustains lung inflammation and remodeling by its interaction with its ligand, receptor for advanced glycation end products (RAGE) (32). In this study, increased HMGB1 release was found in our in vivo and in vitro models of COPD and was enhanced by apoptosis inhibitor Z-VAD but suppressed by necroptosis inhibitor GSK’872. Therefore, necroptosis should be one of the important sources of HMGB1 release in COPD patients. Considering that RAGE also mediates RIP3 phosphorylation (33), we believed that the release of HMGB1 in the process of necroptosis could feed backward to activate RAGE and enhance the RIP3 activation. Other than HMGB1, the release of S100A4 and ATP in the CSE-treated epithelial cells was also suppressed by RIP3 inhibition (Supplemental Figure 3, A and B). The release of DAMPs also skews the polarization of macrophages and suppresses efferocytosis of apoptotic cells (34), which might help explain the higher uptake of necroptotic cells by BMDMs. Additional studies will be required to define the precise molecular mechanism by which necroptotic signals enhance efferocytosis by macrophages.

The traditional necroptosis pathway is initiated by TNF-α stimulation that sequentially activates the RIP1, RIPK3, and MLKL proteins. RIP3 phosphorylates MLKL and translocates p-MLKL to the cell membrane, resulting in cell membrane rupture (11, 12, 35). With respect to CSE-induced necroptosis, we found that CSE-induced MLKL phosphorylation was dependent on RIP3 both in vitro and in vivo. Interestingly, some cancer cells, which do not express RIP3 (17), showed more resistance to CSE-induced cell death, which indicated that the silencing of RIP3 might help the cancer cells escape cell death in smokers. In contrast, inhibition or depletion of RIP1 had less or no protective effect on CSE-induced cell death or CS-induced emphysema in mice in our study (Figure 4A and Figure 6A). This is consistent with a recent study that showed negligible effects on CSE-induced cell death when RIP1 was inhibited in human bronchial epithelial cells (36). RIP3, independent of RIP1, initiation of necroptosis has been reported previously (37, 38); however, the mechanism in general and in COPD has not yet been delineated.

This study, built upon previous reports that have identified necroptosis in COPD (8, 13), shows the essential role of necroptosis in development of emphysema and that inhibition of CS-induced necroptosis by RIP3 inhibitor GSK’872 protects mice from smoking-induced inflammation and subsequent emphysema. Strategies targeting this pathway may lead to novel therapies for COPD. Further studies on necroptosis might be conducted using RIP3- or MLKL-KO mice.

## Methods

### Cell culture and human samples.

The human lung epithelial cell line, Beas-2B, and mouse lung epithelial cell line, MLE-12, were purchased from American Type Culture Collection (ATCC). Cells were grown in DMEM/F12 (Lonza) containing 10% FBS and 1% penicillin/streptomycin. The lung cancer cell lines, A549 and H460, were also purchased from ATCC. Cells were grown in RPMI 1640 containing 10% FBS and 1% penicillin/streptomycin. The HBEs derived from the normal lung (NL) or lung tissue of COPD patients were cultured as previously described (39). The primary organoids of mouse lung were generated using C57BL/6J mice (The Jackson Laboratory) as previously described (40). The BMDMs and AMs derived from BAL were obtained from C57BL/6J mice as described previously (41). The images of organoids were taken using Zeiss inverted microscope.

Human lung tissues were obtained from excess pathologic tissue of patients with COPD after lung transplantation, and normal lung tissue was obtained from donated organs not suitable for transplantation from the Center for Organ Recovery and Education as previously described (42). The demographics of the patients and donors are summarized in Table 1.

### CSE preparation, treatment, and reagents.

CSE was prepared using Kentucky 2R4F research-reference filtered cigarettes (The Tobacco Research Institute, University of Kentucky, Lexington, Kentucky, USA) as previously described (43). One cigarette was used to prepare 10 mL of the cell growth medium, which was regarded as 100% CSE. The epithelial cells were seeded in 12-well plates or 96-well plates 24 hours before CSE treatment. The organoids were subcultured in a 48-well plate for 6 days prior to CSE treatment.

The chemicals used include Vp-16, 3 MA, cycloheximide (MilliporeSigma), Z-VAD (Bachem), TNF-α, Nec-1, and GSK’872 (R&D Systems, Bio-Techne). TNF-α was dissolved in H_2_O. The other agents were dissolved in DMSO (MilliporeSigma) for stock.

### Cell survival assay.

Cell survival assays were performed using the colorimetric method assay (MTS, Promega) and crystal violet staining (MilliporeSigma). Briefly, MLE-12 or Beas-2B cells were seeded in 96-well plates at a density of 1 × 10^4^ cells/well. Following overnight culture, cells were treated with different concentrations of CSE with or without Nec-1, GSK’872, or Z-VAD combination at 37°C for 16 hours. The MTS assay was performed on SpectraMax M2 plate reader (Molecular Devices) according to the manufacturer’s instructions. Each analysis was conducted in triplicate and repeated 3 times. For crystal violet staining, cells were plated at 40%–50% density in 12-well plates, treated with CSE for 16 hours, and then stained with crystal violet staining buffer (3.7% paraformaldehyde, 0.05% crystal violet in distilled water, filtered at 0.45 μm before use).

### Cell death analysis.

Cell death was analyzed by annexin V/PI staining (Invitrogen, Thermo Fisher Scientific) following the manufacturer’s instruction and analyzed by flow cytometry (BD Accuri C6). For apoptosis analysis, the adherent and floating cells after treatment were harvested and resuspended with PBS solution containing 3.7% formaldehyde, 0.5% NP-40, and 10 μg/mL Hoechst 33258 (Invitrogen, Thermo Fisher Scientific). Apoptosis was assessed through microscopic visualization and counting of cells with condensed chromatin and micronucleations as described (44).

### Real-time reverse transcriptase PCR.

Total RNA was isolated from CSE-treated cells using Mini RNA Isolation II Kit (Zymo Research) according to the manufacturer’s protocol. One microgram of total RNA was used to generate cDNA using SuperScript II reverse transcriptase (Invitrogen, Thermo Fisher Scientific). Real-time PCR was performed for *Tnfα*, *Il6*, and *Gapdh* using PowerUp SYBR Green Master Mix (Thermo Fisher Scientific) with previously described conditions (41). The primers were as follows: *Tnfα*: CTGTAGCCCACGTCGTAGC and TTGAGATCCATGCCGTTG; *Il6*: CTTCCATCCAGTTGCCTTCTTG and AATTAAGCCTCCGACTTGTGAAG; *Gapdh*: CGACTTCAACAGCAACTCCCACTCTTCC and TGGGTGGTCCAGGGTTTCTTACTCCTT.

### Western blotting.

Western blotting for the cell lysates was conducted as previously described (41). For the Western blotting of HMGB1 and S100A4 in the cell culture medium, the same number of cells (1 *×* 10^5^) were cultured with the same volume of medium (1 mL). After CSE treatment, the cell culture medium was centrifuged at 500*g* for 5 minutes to get the supernatants. A total of 300 μL of supernatants were mixed with 100 μL 4× loading buffer, and 40 μL of the mixture was loaded on the gel for Western blotting. The following antibodies were used: RIP3 (ab56164), human p-MLKL (S358, ab187091), HMGB1 (ab18256), S100A4 (ab27957) (Abcam), RIP1 (610459, BD Transduction Laboratories), MLKL (MABC604, MilliporeSigma), β-actin (sc-47778, Santa Cruz Biotechnology), mouse p-MLKL (S345, catalog 37333), cleaved caspase-3 (catalog 9661), and GAPDH (catalog 5174) (Cell Signaling Technology). The quantification of Western blot was analyzed by ImageJ software as described on the manufacturer’s website (NIH).

### Transfection and siRNA knockdown.

Transfection of expression constructs and siRNA was performed using Lipofectamine 2000 (Invitrogen, Thermo Fisher Scientific) according to the manufacturer’s instructions. Reverse transcriptase PCR–amplified *RIP3* cDNA from the total RNA of Beas-2B cells was subcloned into a modified pCDNA3.1 plasmid, which contains V5 tag at the C-terminus. siRNA of 200 pmol was transfected into cells 24 hours before CSE treatment. The RIP1 siRNA was purchased from Santa Cruz Biotechnology (sc-36426).

For CRISPR/Cas9-based genome editing, the following sequences were subcloned into pSpCas9(BB)-2A-GFP (Addgene plasmid 48138) targeting mouse *Mlkl*: GCACACGGTTTCCTAGACGC, mouse *Rip3*: GCACAGAAATGGATTGCCCG. The CRISPR/Cas9 KO of target genes was performed as previously described (41).

### Macrophage phagocytosis.

MLE-12 cells were stained with 5 M of CellTrace Far Red dye (Invitrogen, Thermo Fisher Scientific) or CFSE (Invitrogen, Thermo Fisher Scientific) and seeded in 12-well plates (1 *×* 10^5^ cells/well) for flow cytometry or in 8-well Nunc Lab-Tek Chamber Slide System (Thermo Fisher Scientific) for immunofluorescence. After 24 hours, cells were treated with the CSE for 16 hours. BMDMs and AMs were stained with 5 M of CFSE (Invitrogen, Thermo Fisher Scientific) or PE/Cyanine5-CD11b (BioLegend) and afterward added to coculture with MLE-12 cells in 1:1 ratio. Cells were collected and then analyzed by LSRFortessa (BD). Phagocytosis was also analyzed by confocal microscopy using Olympus FluoView 1000 and time-lapse confocal microscopy using Nikon Ti. For the live-cell imaging, cells were imaged every 15 minutes inside a temperature-controlled environmental chamber (Tokai-Hit) atop a Nikon Ti stand equipped with a swept field confocal head, a 60× (1.4NA) optic, and NIS Elements (Nikon Inc.) software.

### ELISA and ATP assay.

The concentrations of HMGB1 and IL-6 in the serum of mice were analyzed by the ELISA kit purchased from R&D Systems, Bio-Techne, as described by the manufacturer. The ATP level in the cell culture medium was analyzed using CellTiter-Glo 2.0 kit (Promega) as described by the manufacturer.

### Mouse exposure to chronic cigarette smoke.

Six-week-old female C57BL/6J mice were purchased from The Jackson Laboratory and raised in the animal facility of the University of Pittsburgh with a 12-hour light/12-hour dark cycle. The mice were exposed to either RA (*n* = 5/group) or CS (4 cigarettes/d, 5 d/wk, *n* = 10/group) as previously described (45). After 2 months of RA or CS exposure, the mice in different groups were injected intraperitoneally with vehicle (4% DMSO), Nec-1 (5 mg/kg in 4% DMSO), or GSK’872 (0.75 mg/kg in 4% DMSO) 1 hour before RA or CS exposure for additional 4 months. Twenty-four hours after the last exposure, mice were sacrificed to obtain the lung tissues and serum. The experiment was repeated twice. One lobe of the lung was dissected for Western blot and flow cytometry. The left lung tissues were inflated at 25 cm H_2_O pressure, fixed in 10% formalin, and embedded in paraffin. Serial midsagittal sections were obtained and stained with modified Gill’s stain (MilliporeSigma). The average chord length and area of lung AMs was analyzed by Scion Image software (version 4.0.2, Scion Corp.) as previously described (46). Cleaved caspase-3, p-MLKL (Cell Signaling Technology), and HMGB1 (Abcam) immunostaining or F4/80 (BD) immunohistochemistry staining was performed on 5 μM formalin-fixed paraffin-embedded lung sections as previously described (16). The flow cytometry analysis was conducted on LSRFortessa (BD) as previously described (47). Cells were stained with various dyes, including CD45-FITC (553080), CD19-APC-Cy7 (561737), CD4-PE (557308) (BD), CD11b-PE-Cy5 (101210), CD8–Pacific Blue (100728), and Ly6G-AF700 (127622) (BioLegend). The results were analyzed with FlowJo software (Tree Star).

### Statistics.

Statistical analyses were carried out using GraphPad Prism IV software (GraphPad Software, Inc.). *P* values were calculated using the Student’s *t* test and 1-way/2-way ANOVA with Tukey’s multiple-comparison test and were considered significant if *P* < 0.05. The means ± SEM were displayed in the figures.

### Study approval.

The use of human subject samples was approved by the University of Pittsburgh Institutional Review Board. All animal experiments were performed in accordance with and with the approval of the Institutional Animal Care and Use Committee of the University of Pittsburgh School of Medicine.

## Author contributions

DC and SDS designed the research. DC, ADG, XL, JW, CLB, GG, and SJS performed the experiments. ADG, CMS, and YZ supplied materials that made this study possible. DC and SDS analyzed the data and wrote the paper. ADG, YZ, and CS gave feedback on the draft paper.

## Supplementary Material

Supplemental data

Supplemental Video 1

Supplemental Video 2

## Figures and Tables

**Table 1:**
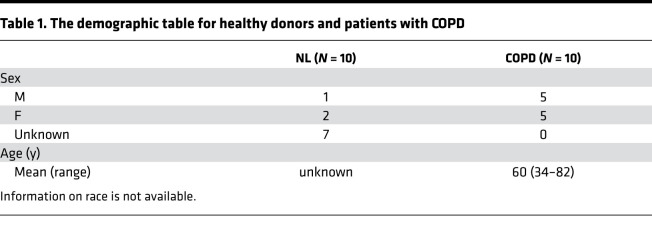
The demographic table for healthy donors and patients with COPD

## Version 1 06/22/2021

Electronic publication
