# Supplementary material for: RIP3-dependent necroptosis contributes to the pathogenesis of chronic obstructive pulmonary disease
Source: JCI Insight. 2021 Jun 22;6(12):e144689. doi: 10.1172/jci.insight.144689 (PMC8262480; doi:10.1172/jci.insight.144689)
Supplement: Supplemental data [file jciinsight-6-144689-s203.pdf]

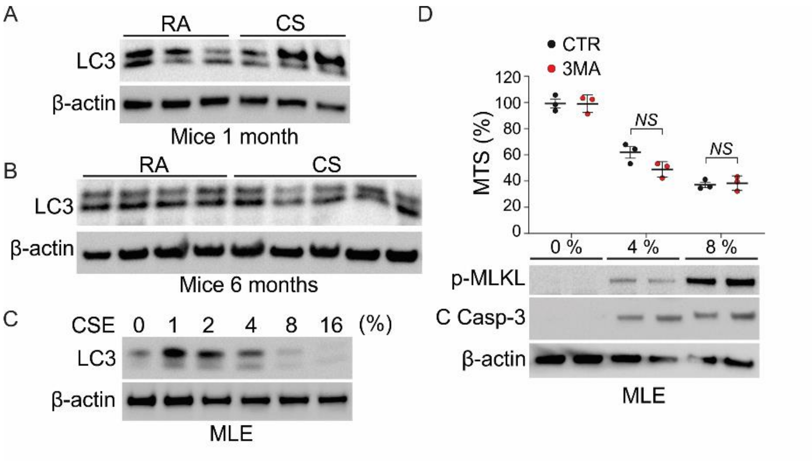

Supplemental Figure 1. CS induced autophagy *in vivo* and *in vitro*. (**A, B**) Western blot analysis of LC3 in the lung tissue from C57BL/6J mice exposed to room air (RA) or cigarette smoking (CS) for 1 month (A) and 6 months (B). (**C**) MLE-12 cells were treated with the indicated concentration of CSE for 16 h. The expression of LC3 in cell lysates was analyzed by western blot. Each experiment was repeated for 3 times. (**D**) MLE-12 cells pretreated with 10 mM 3MA were treated with the indicated concentration of CSE for 16 h. *Upper*, the cell viability was analyzed by MTS assay; *lower*, the expression of indicated proteins in cell lysates was analyzed by western blot. Data represent the means ± SEM. NS, $P>0.05$. Two-way ANOVA with Turkey's multiple comparison test was conducted.

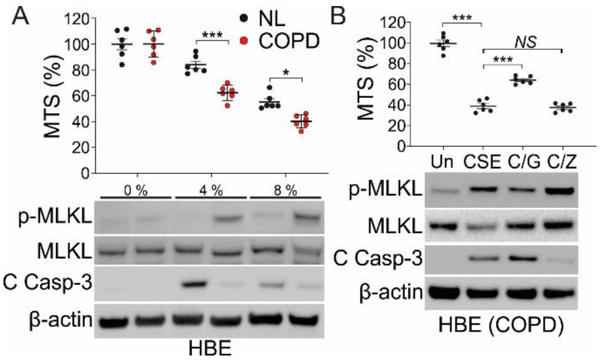

Supplemental Figure 2. CSE induced apoptosis and necroptosis in HBEs. (**A**) HBEs from normal lung (NL) or lung of COPD patients (COPD) were treated with the indicated concentration of CSE for 24 h. *Upper*, the cell viability was analyzed by MTS assay; *lower*, the expression of indicated proteins in cell lysates was analyzed by western blot. (**B**) HBEs (COPD) pre-treated with 10 µM z-VAD (Z), or 5 µM GSK'872 (G) were treated with 8 % CSE for 24 h. *Upper*, the cell viability was analyzed by MTS assay; *lower*, the expression of indicated proteins in cell lysates was analyzed by western blot. The western blot was repeated for 3 times. N=6 for bar graph. Data represent the means ± SEM. NS, $P>0.05$; *****, $P<0.05$; ***, $P<0.001$. Two-way ANOVA with Turkey's multiple comparison test was conducted for (A). One-way ANOVA with Turkey's multiple comparison test was conducted for (B).

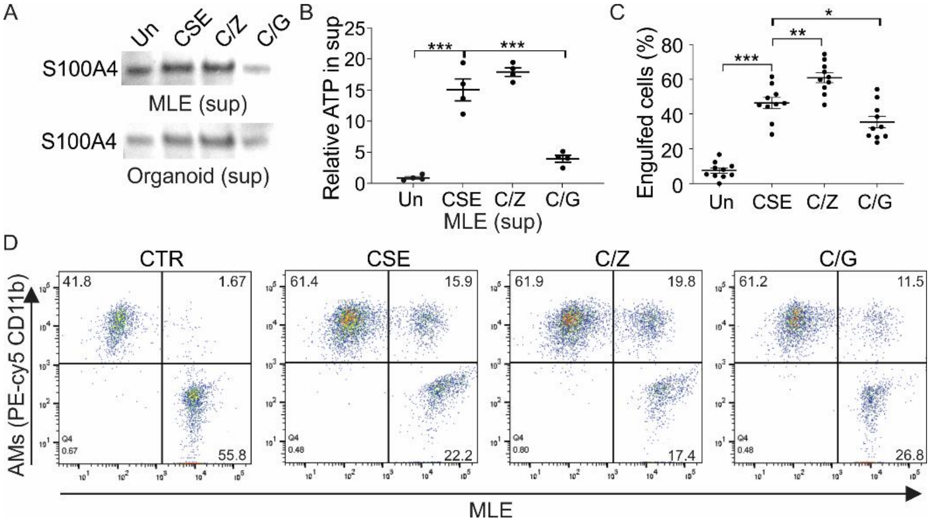

Supplemental Figure 3. Inhibition of necroptosis suppressed DAMPs release and AMs phagocytosis. (**A**) The S100A4 release in the soup of culture medium in MLE-12 (*upper*) and organoids (*lower*) treated with 8 % CSE in combination with 10 µM z-VAD (Z) or 5 µM GSK'872 (G) for 16 h. (**B**) The relative ATP level in the soup of culture medium in MLE-12 treated as in (A) was analyzed by ATP assay. The ATP level was compared with the untreated group. (**C**) MLE-12 cells were treated as in (A) and stained with CellTrace™ Far Red (red), and co-cultured with BMDMs stained with CellTrace™ CFSE (green) for 8 hr. BMDMs phagocytosis was analyzed by confocal microscopy. The percentage of engulfed MLE-12 cells was counted and plotted. (**D**) MLE-12 cells were treated as in (A) and stained with CellTrace™ CFSE (green), and co-cultured with AMs stained with PE-cy5 CD11b for 3 hr. AMs phagocytosis was analyzed by flow cytometry. Representative phagocytosis was shown. Data represent the means ± SEM. *****, *P* <0.05; **, *P*<0.01; *******, *P* <0.001. One-way ANOVA with Turkey's multiple comparison test was conducted.

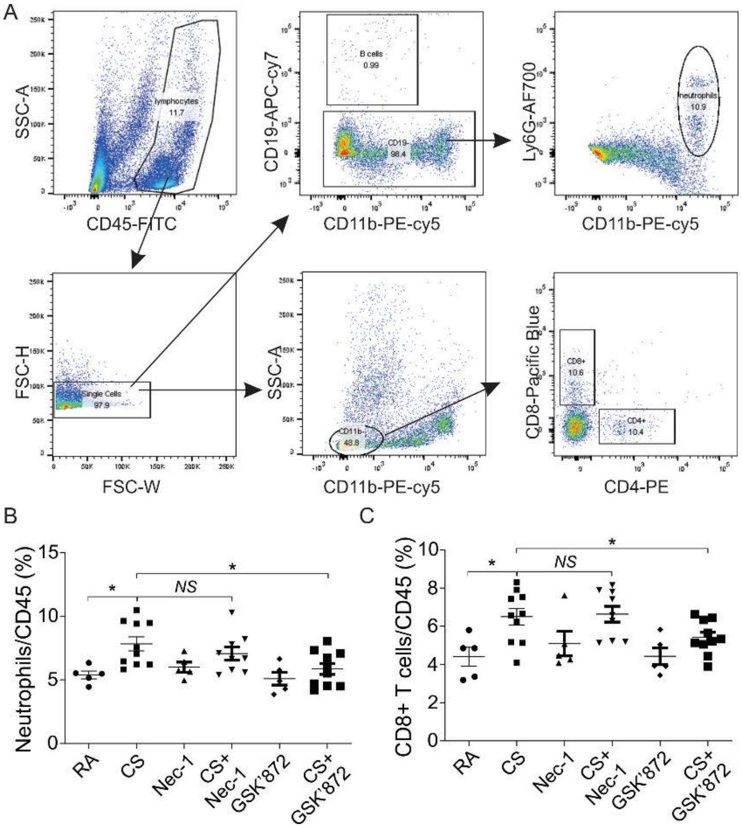

Supplemental Figure 4. GSK'872 suppressed the lung inflammation induced by CS exposure *in vivo*. C57BL/6J mice were exposed to RA (n=5 for each group) or CS (n=10 for each group) for 2 months, and injected with Control vehicle (4 % DMSO), necrostatin-1 (Nec-1, 5 mg/kg), or GSK'872 (0.75 mg/kg) 1 h before CS exposure for additional 4 months. One lobe of lung tissue in each mouse was dissected for flow cytometry analysis of neutrophils and T cells. (**A**) The representative gating of flow cytometry. (**B**) The percentage of neutrophil in the lung lymphocytes was plotted. (**C**) The percentage of CD8+ T cells in the lung lymphocytes was plotted. Data represent the means ± SEM. NS, $P>0.05$; *****, $P<0.05$. One-way ANOVA with Turkey's multiple comparison test was conducted.

Table. 1 The demographic table for healthy donors (NL) and COPD patients

	NL (N=10)	COPD (N=10)
Sex		
M	1	5
F	2	5
Unknown	7	
Age (yr)		
mean(range)	unknown	60 (34-82)

Supplemental Movie 1. Engulfment and digestion of MLE-12 cells (red) treated with CSE in combination with GSK’872 by BMDMs (green).

Supplemental Movie 2. Engulfment and digestion of MLE-12 cells (red) treated with CSE in combination with z-VAD by BMDMs (green).
